# Combination of rAd-p53 in situ gene therapy and anti-PD-1 antibody immunotherapy induced anti-tumor activity in mouse syngeneic urogenital cancer models

**DOI:** 10.1038/s41598-020-74660-2

**Published:** 2020-10-15

**Authors:** Naoto Kunimura, Koichi Kitagawa, Ryota Sako, Keita Narikiyo, Shoko Tominaga, Diosdado S. Bautista, Wei Xu, Masato Fujisawa, Toshiro Shirakawa

**Affiliations:** 1 Department of Advanced Medical Science, Kobe University Graduate School of Science, Technology and Innovation, 7-5-1, Kusunoki-cho, Chuo-ku, Kobe, 650-0017 Japan; 2 Sibiono GeneTech Co. Ltd, Shenzhen, China; 3 Division of Urology, Kobe University Graduate School of Medicine, Kobe, Japan

**Keywords:** Cancer, Drug discovery, Immunology, Oncology, Urology

## Abstract

In this study we undertook a novel combination therapy using rAd-p53 in situ gene therapy and immunotherapy with immune checkpoint inhibitor (ICI) anti-PD-1 antibody for urogenital cancers. Three mouse syngeneic tumor cell lines, TRAMP-C2 (prostate cancer derived from C57BL/6 mice), MBT-2 (bladder cancer derived from C3H mice) and Renca (kidney cancer derived from BALB/c mice) were used in this study. The highest *coxsackie and adenovirus receptor (CAR)* mRNA expression was observed in TRAMP-C2 cells, followed by Renca and then MBT-2 cells. Consistent with the CAR expressions, rAd-p53 at 160 multiplicity of infection (MOI) significantly inhibited the cell proliferation of TRAMP-C2 and Renca cells, but not MBT-2 cells. In in vivo experiments, the combination of intratumoral injections of rAd-p53 (1 × 10^9^ plaque-forming units) every other day and intraperitoneal injections of anti-mouse PD-1 antibody (200 μg) twice a week suppressed tumor growth and prolonged survival compared to rAd-p53 or anti-PD-1 antibody monotherapy in both the TRAMP-C2 and Renca models. Our results encourage the clinical development of combination therapy comprised of in situ gene therapy with rAd-p53 and immunotherapy with an ICI anti-PD-1 antibody for urogenital cancers.

## Introduction

The morbidity and mortality of urogenital cancers including kidney, bladder and prostate cancers in men has tended to increase along with the increase in the aging population^1–3^. Surgery, chemotherapy, radiation, and hormonal therapies, the standard of care for those cancers, often encounter obstacles such as difficulty in resecting the primary tumor in the elderly, acquisition of chemo-resistance, and severe side effects. In response, novel therapeutic approaches like cancer immunotherapy and gene therapy have recently attracted great attention. These novel therapies have the advantages of unique therapeutic effects specific to cancer cells and relatively few side effects.

rAd-p53, one of the most representative cancer gene therapy drugs, is a recombinant replication-deficient adenovirus vector carrying the human *p53* gene^4^. Many previous studies have demonstrated the cytotoxic effects of rAd-p53 on human cancer cells in vitro and in vivo^4–6^. In clinical studies, multiple intratumoral injections of rAd-p53 demonstrated good tumor control of in patients with advanced nasopharyngeal carcinoma, especially when combined with radiotherapy^5^. Despite good local antitumor effects, previous clinical studies of in situ gene therapy, which were performed with intratumoral injections of viral vectors including oncolytic viruses, often failed to achieve a systemic anti-tumor effect or significant improvement of overall survival in patients with multiple distant metastases^6^. In situ gene therapy by local injections with viral vectors thus needs an appropriate companion therapy, such as an immuno-oncology (IO) drug, to induce systemic anti-tumor activity in advanced metastatic tumors.

Recently, several immune checkpoint inhibitors (ICIs), including anti-PD-1 (Programmed cell death 1), anti-PD-L1 (Programmed death-ligand 1) and anti-CTLA-4 (cytotoxic T-lymphocyte-associated protein 4) antibodies have been approved for kidney and urothelial cancers. However, when these ICIs are used as a monotherapy, their response rates are limited to 20 to 30%^7,8^. Obviously, ICIs for urogenital cancers need an adjuvant treatment such as viral gene therapy, which induces innate and adaptive immune responses at the injected site^9^. Indeed, talimogene laherparepvec (T-VEC), a recombinant herpes simplex virus 1 containing the *GM-CSF* (*Granulocyte Macrophage colony-stimulating Factor*) gene, combined with ipilimumab, an anti-CTLA4 antibody, demonstrated greater anti-tumor activity compared to ipilimumab alone in patients with advanced melanoma^10^. Other than oncolytic viruses, a replication-deficient adenoviral vector can also activate cellular immunity. In a clinical study of rAd-HSV-TK, a recombinant replication-deficient adenovirus vector carrying the *HSV-TK (thymidine kinase)* gene, intraprostatic injections of rAd-HSV-TK in patients with prostate cancer induced tumor infiltrating B and T cells^11^.

In the present study, we investigated the synergistic effect of using an anti-PD-1 antibody to enhance the anti-tumor immunity induced by the intratumoral injection of rAd-p53 in experimental syngeneic mouse tumor models of kidney, bladder and prostate cancers in vitro and in vivo.

## Materials and methods

### Cell lines

Three murine cell lines, TRAMP-C2 prostate cancer derived from the C57BL/6 mouse, Renca renal cell carcinoma derived from the BALB/c mouse (purchased from American Type Culture Collection, (ATCC) Manassas, VA) and MBT-2 bladder cancer derived from the C3H/He mouse (purchased from Japanese Collection of Research Bioresources (JCRB), Osaka, Japan) were maintained with Dulbecco’s modified Eagle’s medium (D-MEM; FUJIFILM Wako Pure Chemical Corporation, Osaka, Japan), Roswell Park Memorial Institute 1640 medium (RPMI-1640; FUJIFILM Wako Pure Chemical Corporation) and Eagle's minimal essential medium (E-MEM; FUJIFILM Wako Pure Chemical Corporation). All media were supplemented with 10% fetal bovine serum (FBS; Sigma Aldrich, St. Louis, MO), 100 U/mL penicillin, and 100 μg/mL streptomycin (P/S; Nacalai Tesque, Kyoto, Japan) respectively. All cell lines were grown at 37 °C in a humidified atmosphere of 5% CO_2_.

### Adenovirus vectors

The adenoviral vectors used in this study, rAd-p53 and rAd-LacZ, were constructed as described in our previous study^12^. rAd-p53 is a replication-deficient recombinant adenovirus type 5 vector expressing the cDNA of the wild-type human *p53* gene for the full-length protein, and the rAd-LacZ used is a replication-deficient recombinant adenoviral vector expressing *β-galactosidase* (*LacZ*) gene. rAd-p53 and rAd-LacZ were designed to express the human *p53* gene and *LacZ* gene, under the control of a cytomegalovirus (CMV) immediate-early promoter. The viruses were amplified in HEK293 cells (JCRB) and purified with CsCl_2_ step gradient ultracentrifugation followed by CsCl_2_ linear gradient ultracentrifugation. The purified viruses were dialyzed against a solution containing 10 mM Tris–HCl (pH 7.5), 1 mM MgCl_2_, and 10% glycerol and stored at − 80 °C^13^. Viral particle and biological titers were determined using a standard plaque-forming assay^14^.

### Analysis of mRNA expression levels of coxsackie and adenovirus receptor (CAR)

Cells from each cancer cell line were plated in 6-well culture plate (Corning Inc., Corning, NY) at a density of 1 × 10^6^ cells/well and incubated at 37ºC and 5% CO_2_ for 24 h. Thereafter, cells were collected and total RNAs were extracted using NucleoSpin RNA (TaKaRa, Shiga, Japan). Further, cDNAs were synthesized by reverse transcription of the extracted total RNAs with PrimeScript RT reagent Kit with gDNA Eraser (TaKaRa), and real-time quantitative PCR assays were performed with the primer sets described in Table 1, TB GreenTM Premix Ex TaqTM II (TaKaRa) and Thermal Cycler Dice Real Time System (TaKaRa), and the data analysis was performed by the ΔΔCt method.

**Table. 1:** Primer sequences.

Gene	Sequences (5′ → 3′)
*CAR*	Forward: 5′-CCTGCTGACCGTTCTTGGTA-3'
	Reverse: 5′-CCTTCCCTGCCACCTTGTAA-3'
*p53*	Forward: 5′-CAGCCAAGTCTGTGACTTGCACGTAC-3'
	Reverse: 5′-CTATGTCGAAAAGTGTTTCTGTCATC-3'
*p21*	Forward: 5′-CTTGCACTCTGGTGTCTG-3'
	Reverse: 5′-CCAATCTGCGCTTGGAGTGATAG-3'

### Analysis of mRNA expression levels of p53 and p21in vitro

All three cell lines, TRAMP-C2, MBT-2 and Renca, were plated in 6-well culture plates at a density of 1 × 10^6^ cells/well and incubated at 37ºC and 5% CO_2_ for 24 h. Then the cells were infected with rAd-p53 or rAd-LacZ at multiple concentrations of multiplicity of infection (MOI) and further incubated for 48 h. Thereafter, the cells were collected. and the total RNAs were isolated and analyzed by the real-time quantitative PCR to quantify the expression of *p53* and *p21* mRNAs as described above.

### Analysis of protein levels of p53 in vitro

Cells from each cancer cell line were plated in 6-well culture plate at a density of 5 × 10^5^ cells/well and incubated at 37ºC and 5% CO_2_ for 24 h. Then the cells were infected with rAd-p53 or rAd-LacZ at 40 MOI for 48 h. Cells were washed and lysed in 8 M urea containing cOmplete Protease Inhibitor Cocktail (Roche, Basel, Switzerland). Each sample was added into sample buffer (Nacalai Tesque, Kyoto, Japan) and heated at 95 °C for 5 min. The samples were separated by SDS-PAGE and transferred to a Polyvinylidene difluoride membrane. After blocking with Blocking One P (Nacalai Tesque) followed by washing, the membranes were incubated for 1 h at room temperature with anti-p53 (Abcam, Cambridge, UK) or anti-β-actin (Santa Cruz Biotechnology, Dallas, TX). After another washing, membranes were incubated for 1 h with HRP-conjugated secondary antibodies. Antibody binding to proteins was detected by enhanced chemiluminescence.

### Cell proliferation assay

Cells were plated in 96-well culture plates (Corning) at a density of 1 × 10^3^ cells/well. After the cells adhered, they were infected with rAd-p53 or rAd-LacZ at 10, 40 and 160 MOIs. Six days after the infection, the viable cell density was measured by Alamar Blue assay (Invitrogen, Waltham, MA).

### Flow cytometry analysis for PD-L1 expression

The expressions of PD-L1 on the cell surface were assessed by flow cytometry. The cells were plated in 6- well culture plates (Corning) at a density of 1 × 10^6^ cells/well and incubated at 37ºC and 5% CO_2_ for 24 h. Then 10 ng/mL of IFN-γ was added to the cells for stimulation, and the cells were incubated for 48 h. After infection with rAd-p53 or rAd-LacZ at 40 MOI, the cells were incubated for 48 h, harvested, washed with PBS, and supplemented with 20 µL of Blocking One Histo (Nacalai Tesque). After the incubation for 10 min at room temperature, the cells were labeled with antibody against phycoerythrin (PE)-conjugated anti-human CD274 (BioLegend, San Diego, CA) at 4ºC for 30 min. After the cells were washed again with PBS, fluorescence-activated cell sorting and analysis were performed using Explore Guava easyCyte Benchtop Flow Cytometers (Merck Millipore, Darmstadt, Germany).

### Animal experiments

The TRAMP-C2 mouse syngeneic prostate cancer model and Renca mouse syngeneic kidney cancer model were employed for in vivo experiments to investigate the efficacy of combining rAd-p53 and anti-mouse PD-1 antibody. Male C57BL/6 (TRAMP-C2) or BALB/c (Renca) mice 6 weeks of age were purchased from CLEA Japan, Inc. (Tokyo, Japan). The mice were housed for 7 days and given free access to food and water. To induce subcutaneous syngeneic tumors, we injected 2 × 10^6^ TRAMP-C2 or Renca cells in a total volume of 140 µL of 1/1 (v/v) PBS/ Matrigel Martrix Basement Membrane HC (Corning) in the right flank of the mice. Twenty-five tumor bearing mice each for TRAMP-C2 or Renca were randomly assigned to 5 treatment groups (n = 5) as follows; rAd-p53 + anti-PD-1 antibody, rAd-LacZ + anti-PD-1 antibody, PBS + anti-PD-1 antibody, rAd-p53 alone, rAd-LacZ alone and PBS. For the TRAMP-C2 model, 1 × 10^9^ plaque-forming units (pfu)/50 µL of rAd-p53 or rAd-LacZ was injected intratumorally every other day (for a total of 12 times) starting when tumor growth was confirmed 53 days after the tumor inoculation. Three days after starting injection of rAd-p53, 200 µg of anti-mouse PD-1 antibody (Bio X Cell Inc, West Lebanon, NH) was intraperitoneally injected into mice twice a week (6 times in total). For the Renca model, 1 × 10^9^ pfu/50 µL of rAd-p53 or rAd-LacZ was injected intratumorally every other day (for a total of 8 times) starting when tumor growth was confirmed, 12 days after the tumor inoculation. Three days after starting injection of rAd-p53, 200 µg of anti-mouse PD-1 antibody was intraperitoneally injected into mice twice a week (for a total of 4 times). The tumor volumes were determined every third day by measuring in two dimensions, length (L) and width (W), and calculating volume as (W^2^ × L)/2. The endpoints were > 25 mm of tumor length for TRAMP-C2 or > 2500 mm^3^ of tumor volume for Renca, respectively.

### Immunohistochemical staining

Another set of mice was inoculated with Renca and treated as described above. Mice were euthanized and tumor tissues were resected and fixed with paraformaldehyde. Paraffin embedded tumor tissue sections Renca were deparaffinized and rehydrated. Antigen retrieval was performed in Bond epitope retrieval buffer (pH6.0 for CD8a, pH9.0 for CD4; Leica Microsystems, Wetzlar, Germany) at 98 °C for 20 min. Immunohisochemical staining was performed in automatic tissue processor (Leica Microsystems Bond) according to manufacturer’s standard protocol. Briefly, tissue sections were incubated at RT for 15 min with rabbit anti-mouse CD4 antibody (1:1000, Abcam, Cambridge, UK) or rabbit anti-mouse CD8a antibody (1:400, Cell Signaling Technology Japan, Tokyo, Japan). After washing, sections were incubated with horse radish peroxidase-conjugated secondary antibodies. After washing, sections were incubated with 3,3′-diaminobenzidine and were counterstained with hematoxylin. Resulting tissue slides were observed under microscope BZ-X710 (Keyence, Osaka, Japan).

### Flowcytometry for tumor infiltrating lymphocytes (TILs) and tumor cells in Renca tumor

Resected tumors were mechanically homogenizedand strained to make single cell suspension in 10 mM EDTA-PBS. Cells were blocked with anti-CD16/32 for 20 min on ice and stained with PerCP-anti-CD3, FITC-anti-CD4, and APC-anti-CD8 and PE-anti-CD107a antibodies for 30 min on ice. Then, cells were washed and measured by flowcytometry. Then, the washed cells were fixed and permeabilized with BD Fixation/Permeabilization solution. Washed and stained cells were measured by flow cytometer. The PD-L1 expressions in homogenized tumor cells in PBS and rAd-p53 groups were also measured by flow cytometer as described above for flow cytometry analysis for PD-L1expression.

### Statement

All experiments and methods were performed in accordance with relevant guidelines and regulations, and all experimental protocols were approved by the committees of the Kobe University Graduate School of Medicine. Specifically, the animal experimental design and procedure were reviewed and approved by the institutional ethics and animal welfare committees of the Kobe University Graduate School of Medicine.

### Statistical analysis

Comparisons between multiple groups were performed by one-way ANOVA followed by the Tukey–Kramer method. Differences among experimental groups were considered significant when *p* < 0.05. Survival between groups was analyzed by the log-rank (Mantel-Cox) test and adjusted by Bonferroni-corrected p-value (*p* < 0.0083) for Kaplan–Meier curves.

### Ethical standards

All aspects of the experimental design and procedures involving animals were reviewed and approved by the institutional ethics and animal welfare committees of the Kobe University Graduate School of Medicine.

## Results

### TRAMP-C2 cells expressed the highest level of CAR mRNA among the three cell lines

**Figure 1:**
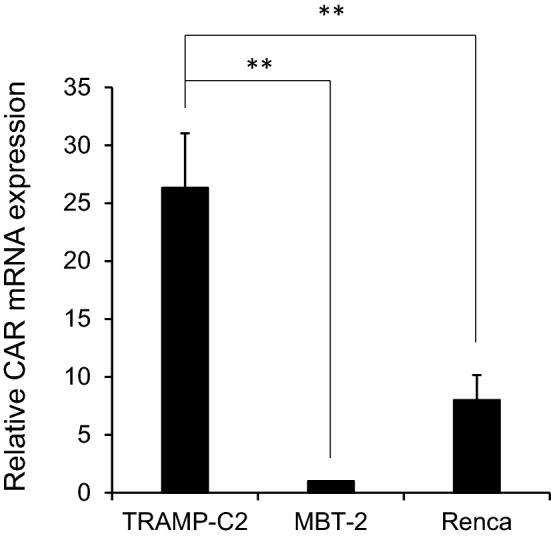
The relative mRNA expression levels of *CAR* gene in TRAMP-C2, MBT-2, and Renca cell lines. Relative mRNA levels were determined by real-time PCR assay. Each point represents the average of triplicate relative values normalized by the MBT-2 cells; *bars* ± SD, (**p < 0.01). β-Actin was used as an endogenous RNA control to normalize for differences in the amount of total RNA. Each data was obtained from three different experiments.

We evaluated the relative mRNA expression levels of *coxsackie and adenovirus receptor* (*CAR*) gene in the TRAMP-C2, MBT-2 and Renca cell lines by real-time PCR assay. As shown in Fig. 1, TRAMP-C2 expressed significantly higher mRNA expression levels of *CAR* compared to the other cell lines (*p* < 0.01), and Renca expressed significantly higher *CAR* mRNA compared to MBT-2 (*p* < 0.01).

### rAd-p53 induced overexpression of p53 gene and protein in all three cell lines, but induced overexpression of p21 gene only in TRAMP-C2

**Figure 2:**
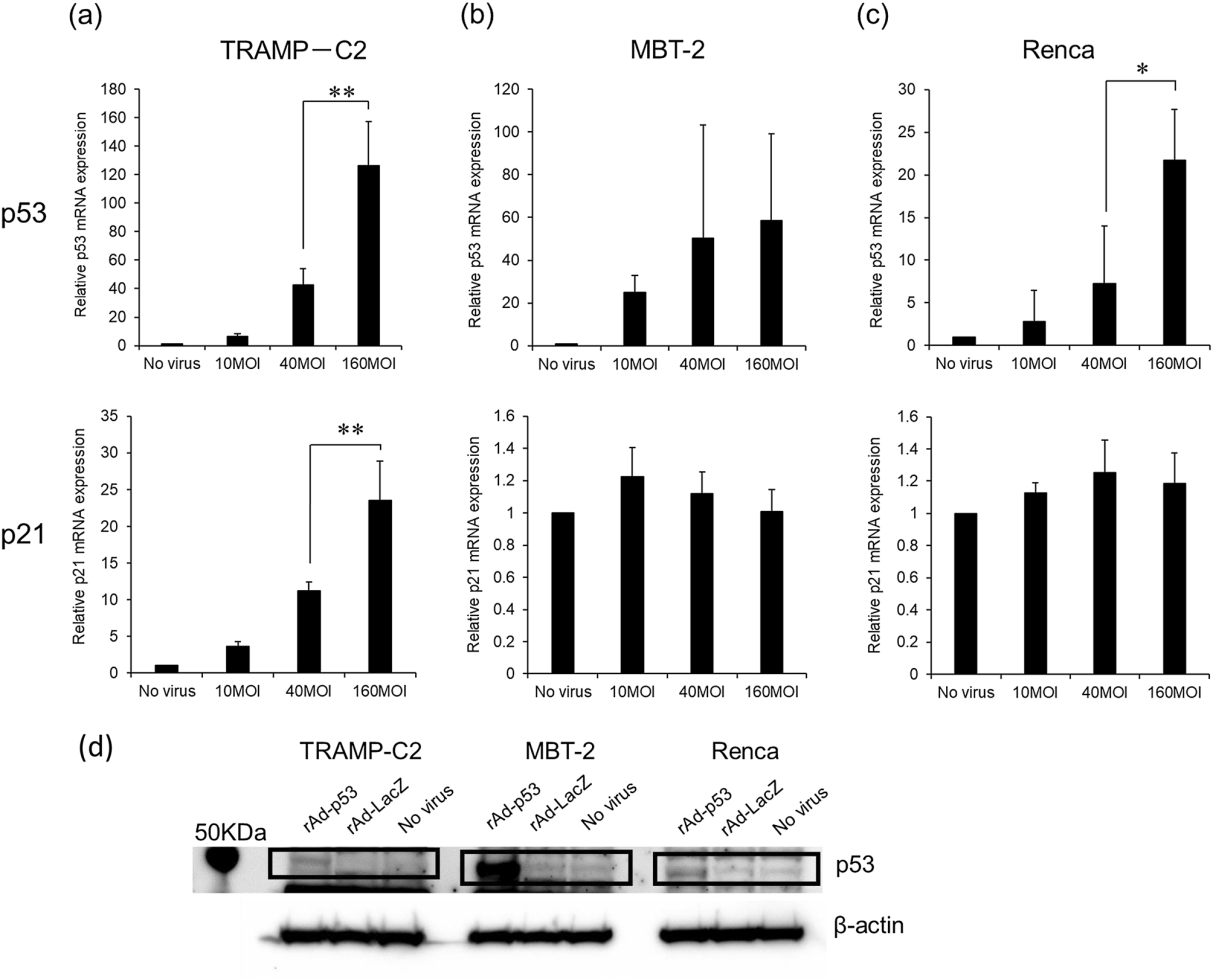
The relative mRNA expression of *p53* and *p21* genes and protein expression of p53 in TRAMP-C2, MBT-2, and Renca cell lines. Relative mRNA expression of *p53* and *p21* in TRAMP-C2 (**a**), MBT-2 (**b**), and Renca (**c**), was determined by real-time PCR after infection with rAd-p53 at 10, 40 and 160 MOI. Each data point represents the average of triplicate relative values normalized by the value when cells were treated with no virus from three different experiments: *bars* ± SD, (*p < 0.05, ** p < 0.01). β-Actin was used as an endogenous RNA control to normalize for differences in the amount of total RNA. (**d**) Over-expressions of p53 protein were observed in all three cell lines infected with rAd-p53 by Western blotting. The original images of the Western blotting gels were shown in ‘supplementary file’.

We conducted real-time PCR assays to investigate whether rAd-p53 could induce overexpression of *p53* and *p21* genes sequentially in a dose-dependent manner. Correlated to the expression levels of *CAR*, increases of *p53* mRNA levels depending on rAd-p53 doses were observed only in TRAMP-C2 (*p* < 0.01) and Renca (*p* < 0.05), but not in MBT-2 cell lines (Fig. 2a,b,c). Although rAd-p53 significantly increased the expression levels of *p53* mRNAs at 10, 40, and 160 MOIs compared to no virus infection in MBT-2, no dose-dependency was observed (Fig. 2b). Also, the sequential increase of *p21* mRNA in a dose-dependent manner was observed only in the TRAMP-C2 cell line and not in the other cell lines (*p* < 0.01) (Fig. 2a,b,c). In addition, the western blotting analysis revealed that rAd-p53 could induced the overexpression of p53 protein in all three cells (Fig. 2d). The original images of the Western blotting gels were shown in ‘supplementary file’.

### rAd-p53 significantly inhibited the cell proliferation of TRAMP-C2 and Renca cell lines in vitro

**Figure 3:**
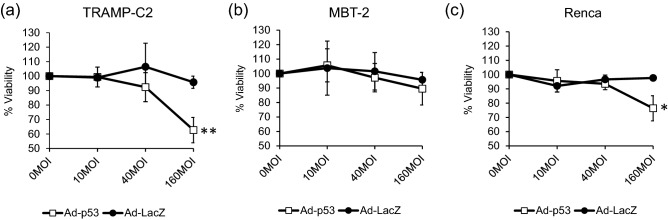
Cell cytotoxicity with rAd-p53 in TRAMP-C2, MBT-2, and Renca cell lines. Cell cytotoxicity was determined after infection with rAd-p53 at 10, 40 and 160 MOI in TRAMP-C2 (**a**), MBT-2 (**b**), and Renca (**c**). rAd-LacZ was used as a control vector. Each data point represents the average of triplicate values normalized by the value when cells were treated with no virus from three different experiments: *bars* ± SD, (*p < 0.05, ** p < 0.01).

We evaluated the in vitro inhibitory effects of rAd-p53 on cell proliferation of TRAMP-C2, MBT-2 and Renca cell lines by Alamar blue assay. As shown in Fig. 3, rAd-p53 significantly inhibited the cell proliferation of TRAMP-C2 (*p* < 0.01) and Renca (*p* < 0.05) cell lines at 160 MOI on day 6 compared to rAd-LacZ treated cells, but not the cell proliferation of MBT-2.

### IFN-γ increased PD-L1 expression in vitro in all three cells

**Figure 4:**
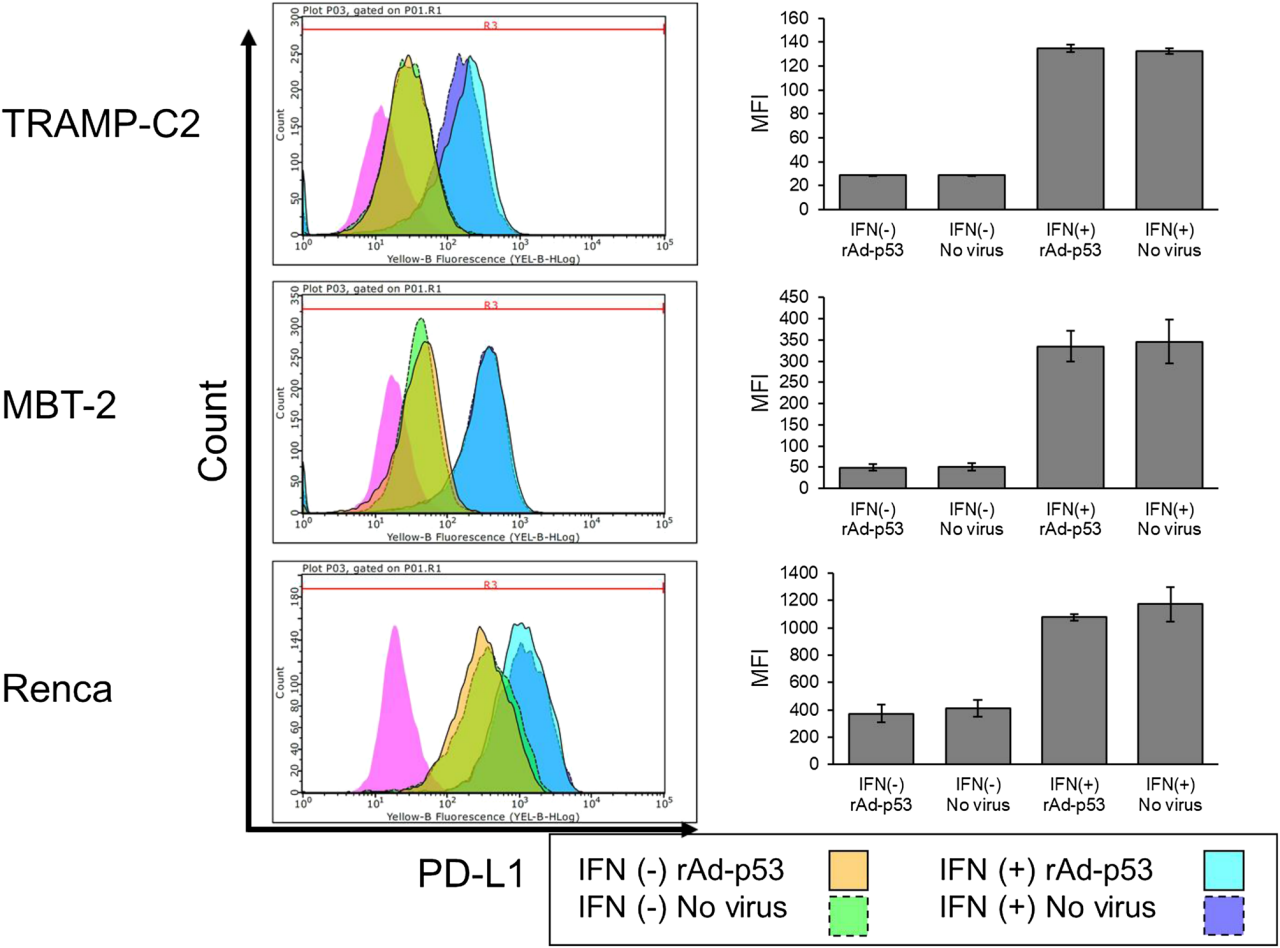
Expression of PD-L1 after infection with rAd-p53 in TRAMP-C2, MBT-2, and Renca cell lines. TRAMP-C2, MBT-2, and Renca were cultured in the presence of 10 ng/mL IFN-γ and infected with rAd-p53 at 40 MOI. The expressions of PD-L1 were shown in each histogram. The expression of PD-L1 was shown as mean fluorescent intensity (MFI). The fold of the expression of PD-L1 were calculated by the MFI at IFN (+) with rAd-p53 /IFN (−) with rAd-p53 and compared with the MFI with no virus. The averages of mean fluorescent intensity (MFI) were obtained from three different experiments.

To investigate the effect of rAd-p53 infection on PD-L1 expression in the three cell lines, we evaluated the expression levels of PD-L1 with IFN-γ stimulation in rAd-p53 infected or non-infected TRAMP-C2, MBT-2 and Renca cells by flow cytometry. As shown in Fig. 4, IFN-γ substantially increased PD-L1 expressions in all three cell lines, but rAd-p53 did not change the PD-L1 expressions in TRAMP-C2 (from MFI: mean fluorescence intensity, 132.5 ± 2.4 to MFI 134.7 ± 3.0), in Renca (from MFI 1174.0 ± 104.3 to MFI 1078.6 ± 19.4), and in MBT-2 (from MFI 345.7 ± 51.4 to MFI 335.3 ± 35.7) in vitro (Fig. 4).

### Combination of rAd-p53 and anti-PD-1 antibody induced synergistic anti-tumor effects in TRAMP-C2 and Renca syngeneic tumor models in vivo

**Figure 5:**
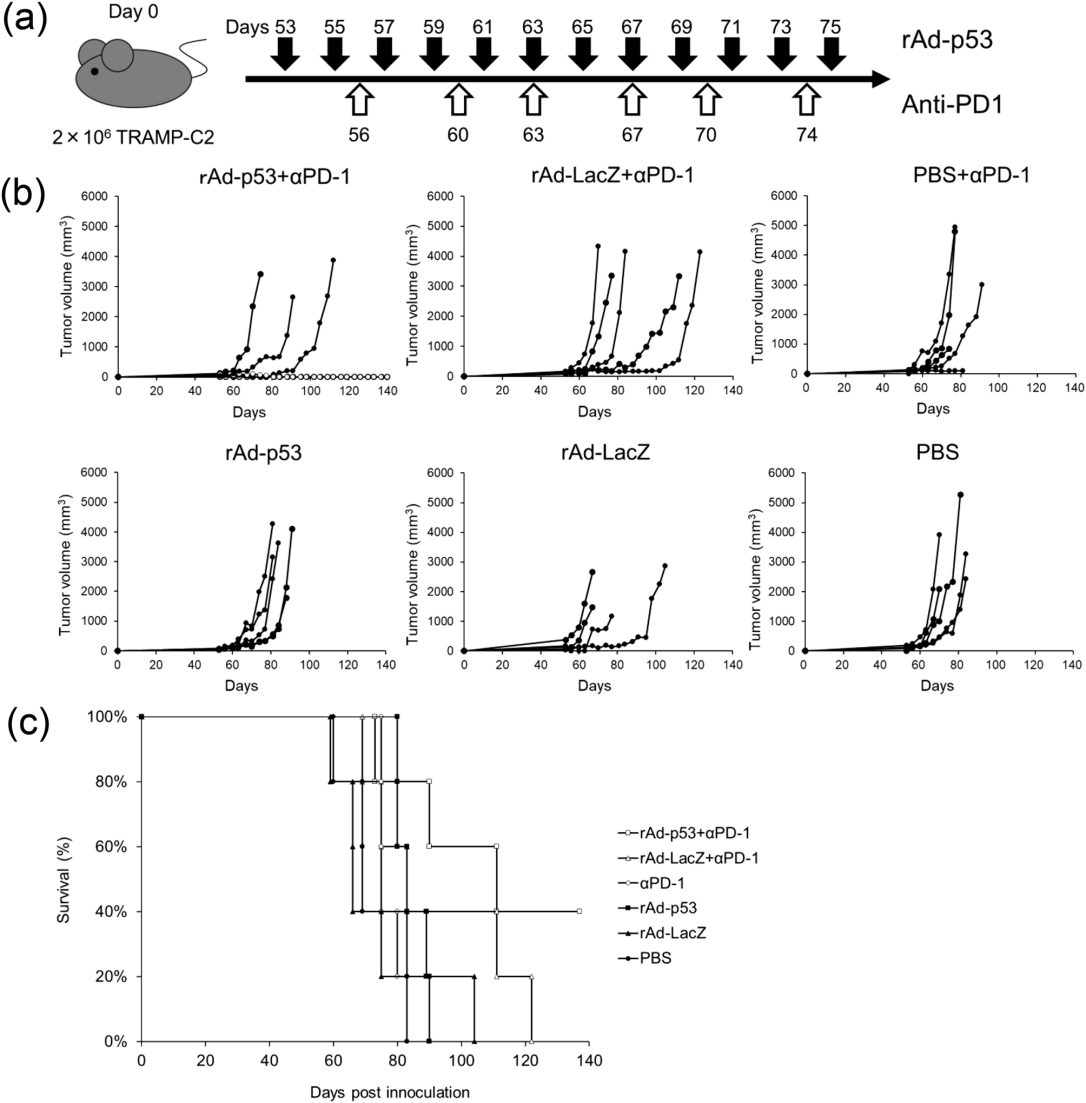
Combination therapy with rAd-p53 and anti-PD-1 antibody in the TRAMP-C2 syngeneic tumor model. Two million TRAMP-C2 cells were subcutaneously inoculated into C57BL/6 mice at day 0. 1 × 10^9^ PFU of rAd-p53, rAd-LacZ or PBS were intratumorally injected every other day starting 53 days after tumor inoculation (n = 5). Anti-PD-1 antibody was intraperitoneally injected twice a week (6 times total) starting 56 days after tumor inoculation: (**a**) a schematic drawing of the treatment. (**b**) Tumor growth curves of individual mice in each treatment group. Open circles are mice alive at day 140. (**c**) Kaplan–Meier survival curve post tumor inoculation. The combination therapy of rAd-p53 and anti-PD-1 antibody substantially prolonged survival compared to other treatments.

**Figure 6:**
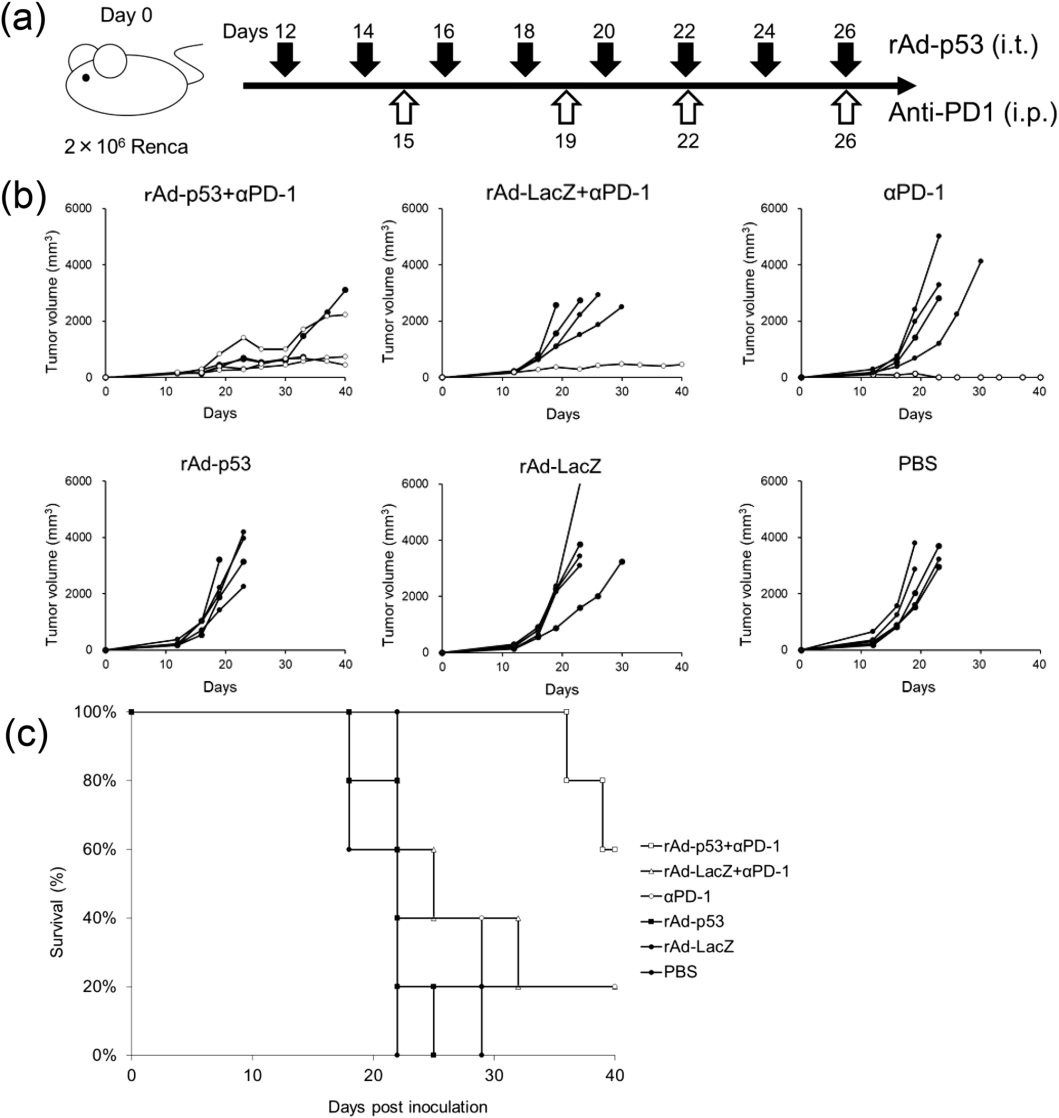
Combination therapy with rAd-p53 and anti-PD-1 antibody in the Renca syngeneic tumor model. Two million Renca cells were subcutaneously inoculated into Balb/c mice at day 0. 1 × 10^9^ PFU of rAd-p53, rAd-LacZ or PBS were intratumorally injected every other day starting 12 days after tumor inoculation (n = 5). Anti-PD-1 antibodies were intraperitoneally injected into mice twice a week (6 times total) starting 15 days after tumor inoculation: (**a**) a schematic drawing of the treatment. (**b**) Tumor growth curves of individual mice in each treatment group. Open circles are mice alive at day 140. (**c**) Kaplan–Meier survival curve post tumor inoculation. The combination therapy of rAd-p53 and anti-PD-1 antibody substantially prolonged survival compared to other treatments.

To investigate the synergistic anti-tumor efficacy of combining rAd-p53 and anti-PD-1 antibody in vivo, we employed TRAMP-C2 and Renca syngeneic tumor models for in vivo experiments because significant in vitro cytotoxicity by rAd-p53 was observed only in the TRAMP-C2 and Renca cell lines, not in MBT-2. In the in vivo studies, the combination of rAd-p53 and anti-PD-1 antibody (a schematic drawing of the combination treatment showed in Figs. 5a,6a) suppressed TRAMP-C2 and Renca tumor growth compared to the other treatment groups after tumor inoculation (Figs. 5b,6b). In TRAMP-C2 model, two out of five mice treated with combination of rAd-p53 and anti-PD-1 antibody were alive at day 140, whereas all mice were dead in the other treatment groups at day 140 (Fig. 5b). In Renca model, three out of five mice treated with combination of rAd-p53 and anti-PD-1 antibody were alive at day 40, whereas one out of five mice treated with rAd-LacZ and anti-PD-1 antibody, or anti-PD-1 antibody alone was alive, and all mice were dead in the other treatment groups at day 40 (Fig. 6b). Also, the combination of rAd-p53 and anti-PD-1 antibody substantially prolonged the survival of mice bearing TRAMP-C2 (Fig. 5c) or Renca (Fig. 6c) tumors compared to the other treatment groups.

### Combination of rAd-p53 and anti-PD-1 antibody induced tumor infiltrating T cells and rAd-p53 increased PD-L1 expression on tumor cells in vivo

**Figure 7:**
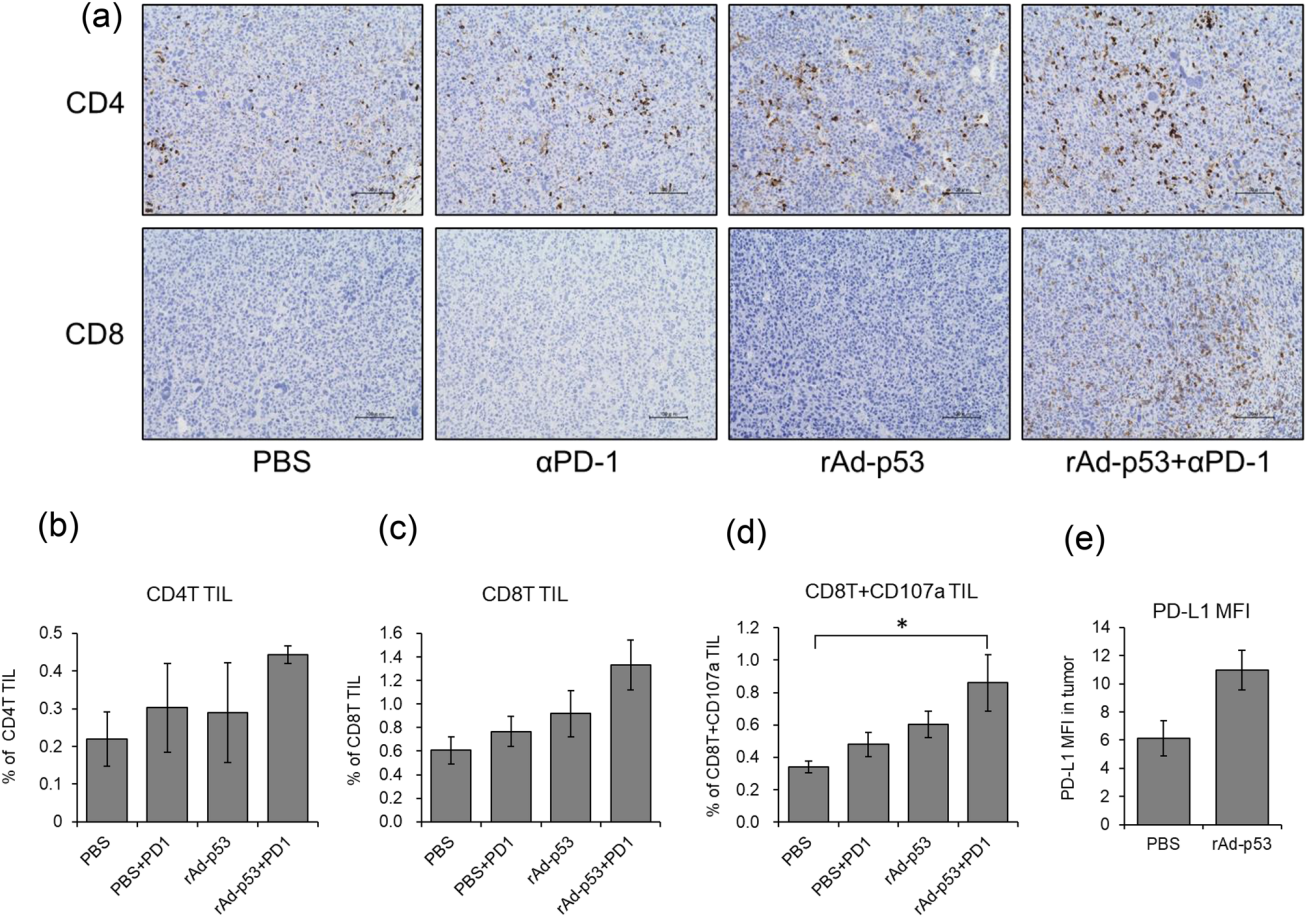
Tumor infiltrating lymphocytes were induced by combination of rAd-p53 and anti-PD1 antibody in Renca tumor. After treatment with PBS, anti-PD1 antibody, rAD-p53, or rAd-p53 and anti-PD1 antibody, Renca tumor tissues were resected and stained with anti-CD4 antibody or anti-CD8 antibody to detect tumor infiltrating lymphocytes (TILs). (**a**) Representing images from each treatment group were shown (200 ×). (**b**, **c**, and **d**) flowcytometry analysis for tumor infiltrating CD4T (**b**), CD8T (**c**) and CD107a-positive CD8T (**d**) cells in Renca tumor. Data were obtained from three different experiments using different mice: *bars* ± SE, (*p < 0.05). (**e**)The expression of PD-L1 in Renca tumor after treatment with PBS or rAd-p53 were shown. Data were obtained from three different experiments using different mice: *bars* ± SE.

As the result of immunohistochemical staining, we found remarkable numbers of CD4 and CD8 positive lymphocytes were infiltrated into the Renca tumor tissues in rAd-p53 with anti-PD-1 group (Fig. 7a). These results suggested that combination therapy of rAd-p53 with anti-PD-1 induced tumor-infiltrating CD4T and CD8T cells in tumor tissues in syngeneic renal cancer model. Furthermore, flow cytometric analysis revealed that the combination of rAd-p53 with anti-PD-1 induced the highest number of tumor infiltrating CD4T and CD8T cells in Renca tumor tissues among all treatment groups (Fig. 7b,c). The number of CD107a-positive CD8T cells was significantly increased in the combination of rAd-p53 and anti-PD-1 antibody compared to PBS group (Fig. 7d). The CD107a is a lysosomal-associated membrane protein, which is a degranulation marker for CTL, and these findings suggested that the combination of rAd-p53 and anti-PD-1 antibody induced the activated CTLs. In addition, rAd-p53 injection substantially increased PD-L1 expression in in vivo tumor tissues compared to PBS injection (Fig. 7e).

## Discussion

In the present study, we explored a novel combination therapy combining in situ viral gene therapy and cancer immunotherapy using ICIs for the treatment of urogenital cancers. Recently, a number of clinical studies have been reported in non-urogenital cancers, including T-Vec combined with ipilimumab (ant-CTLA-4 antibody)^10^, Cavatak (oncolytic coxsackievirus) or Reolysin (oncolytic reovirus) combined with pembrolizumab (anti-PD-1 antibody) and other combinations^15^. Also, a preclinical study of a replication-deficient adenoviral vector carrying *HSV-tk* gene combined with an anti-PD-1 antibody demonstrated a great anti-tumor activity in syngeneic mouse glioblastoma models^16^. In our experiments, the feasibility of combination therapy comprised of in situ gene therapy with rAd-p53 and anti-PD-1 antibody was investigated in mouse syngeneic urogenital cancer models of kidney, bladder and prostate cancers.

Among several serotypes of adenovirus, serotype 5 is most commonly used for the construction of adenoviral vectors. Previously, CAR encoding a 46-kDa protein was identified as the receptor for group C adenovirus including serotypes 2 and 5, and the expression of CAR on the cell surface is strongly correlated with the infectivity of adenovirus type 5^17^. Despite the correlation between CAR and infectivity of adenovirus type 5, previous reports regarding CAR expressions in cancer types are controversial and some types of cancer do not express CAR^18^. Therefore, we first confirmed the CAR expressions in the three mouse tumor cell lines used in this study. TRAMP-C2 cells expressed the highest level of *CAR* gene among the all cell lines, and MBT-2 cells expressed the lowest level of *CAR* gene (Fig. 1). Along with CAR expression, TRAMP-C2 showed the highest *p53* gene transduction by rAd-p53 among the three cell lines and sequentially expressed the highest *p21* gene level (Fig. 2). The p21 protein is a downstream protein of p53 and is considered to be a universal inhibitor of cell cycle progression. As for the in vitro cytotoxicity of rAd-p53 in the three cell lines, rAd-p53 significantly inhibited the cell growth of TRAMP-C2 and Renca but not MBT-2 (Fig. 3). These results confirmed that the efficacy of adenovirus type 5 vector could well be correlated with CAR expression in target cancer cells. To overcome the loss of CAR expression in target cancer cells, fiber-substituted adenovirus type 5 vectors containing the fiber knob and shaft of adenovirus type 35, which can bind to CD46 receptor on the surface of human cells, were developed^19^. Also, chimeric adenoviruses containing the fiber knob and shaft of type 3 and/or 11, which bind to CD46 and Desmoglein-2, have been developed^20,21^.

It is well known that in situ viral gene therapy activates both innate and adaptive immunities through Th1 responses in the tumor microenvironment to inhibit tumor growth^22–24^. However, in situ viral gene therapy also induces PD-L1 expression on the tumor cells^16^. In the present study, rAd-p53 did not increase PD-L1 expression on the cell surface of the three tumor cell lines, in vitro (Fig. 4). Nonetheless, we confirmed the substantial increase of PD-L1 expression by rAd-p53 injection in in vivo Renca tumors (Fig. 7e). Thus, it is a reasonable strategy to combine in situ viral gene therapy with PD-1/PD-L1 inhibitors. In addition, p53 is directly involved in the regulation of antigen presentation through the major histocompatibility complex (MHC) I pathway for cytotoxic T lymphocytes (CTL)^25^.

In the in vivo experiments, we investigated anti-tumor activity in TRAMP-C2 and Renca mouse syngeneic tumor models, in which the in vitro cytotoxicity of rAd-p53 was confirmed. The results showed that the combination of intratumoral injections of rAd-p53 and intraperitoneal injections of anti-PD-1 antibody demonstrated the highest tumor growth inhibitory effects and the longest survival periods among the all treatment groups in both tumor models (Figs. 5, 6). Despite the high in vitro cytotoxicity of rAd-p53 in TRAMP-C2 cells (Fig. 3), the in vivo efficacy of combination of rAd-p53 and anti-PD-1 antibody in Renca tumor was superior to that in TRAMP-C2 (Fig. 5 and 6). This finding suggested that the anti-tumor immune response more greatly affected the in vivo efficacy of this combination therapy than the cytotoxicity of rAd-p53. Furthermore, the combination of rAd-p53 and anti-PD-1 antibody induced a significantly higher TILs including activated CTLs, which is CD8 and CD107a positive T cells. The CD107a is a degranulation maker for CTL releasing granzyme and perforin. At this moment, no ICIs have been approved for prostate cancer, and most clinical trials, including a phase III study with ICIs, CTLA-4 and PD-1/PD-L1 inhibitors, are disappointing^26^. One of the reasons for poor response to ICIs in prostate cancer is the low level of tumor infiltrating lymphocytes (TILs) based on a relatively low tumor mutational burden (TMB)^26^. Following the US Food and Drug Administration (FDA) approval of nivolumab, an anti-PD-1 antibody for kidney cancer in 2015, other ICIs including ipilimumab (anti-CTLA-4), pembrolizumab (anti-PD-1), and avelumab (anti-PD-L1) were also approved in combinational use with other ICIs or axitinib (a VEGF receptor tyrosine kinase inhibitor)^27^. However, a novel combination therapy using ICIs is still being sought out for better clinical outcomes in advanced kidney cancer. In such a combinational strategy, in situ viral gene therapy could forcibly induce the TILs and increase the sensitivity to ICIs.

In conclusion, the present study demonstrated that the combination of in situ gene therapy with rAd-p53 and immunotherapy with an anti-PD-1 antibody induced good anti-tumor activity in mouse TRAMP-C2 and Renca syngeneic tumor models, which both express CAR. Although improvements in the universal infectivity of adenovirus type 5 vector are required, our results encourage the clinical development of a combination therapy comprised of in situ viral gene therapy and immunotherapy with ICIs for urogenital cancers.

## Supplementary information

Supplementary Information.
