# Supplementary material for: Combination of rAd-p53 in situ gene therapy and anti-PD-1 antibody immunotherapy induced anti-tumor activity in mouse syngeneic urogenital cancer models
Source: Sci Rep. 2020 Oct 15;10:17464. doi: 10.1038/s41598-020-74660-2 (PMC7562933; doi:10.1038/s41598-020-74660-2)

Combination of rAd-p53 in situ gene therapy and anti-PD-1 antibody immunotherapy induced anti-tumor activity in mouse syngeneic urogenital cancer models

Naoto Kunimura[1], Koichi Kitagawa[1], Ryota Sako[1], Keita Narikiyo[1], Shoko Tominaga[1], Diosdado S. Bautista[2], Wei Xu[2], Masato Fujisawa[3], Toshiro Shirakawa[1, 3]

Affiliations

1. Department of Advanced Medical Science, Kobe University Graduate School of Science, Technology and Innovation, Kobe, Japan
2. Sibiono GeneTech Co. Ltd., Shenzhen, China
3. Division of Urology, Kobe University Graduate School of Medicine, Kobe, Japan

***Corresponding author:**

Toshiro Shirakawa MD, PhD.

Professor

Department of Advanced Medical Science, Kobe University Graduate School of Science, Technology and Innovation, 7-5-1, Kusunoki-cho, Chuo-ku, Kobe, 650-0017, Japan

Tel.: +81-78-382-5681

Fax: +81-78-382-5715

E-mail: toshiro@med.kobe-u.ac.jp

Running title: Combination of rAd-p53 and ICI for urogenital cancers

Original Image of P53 Western Blotting gel

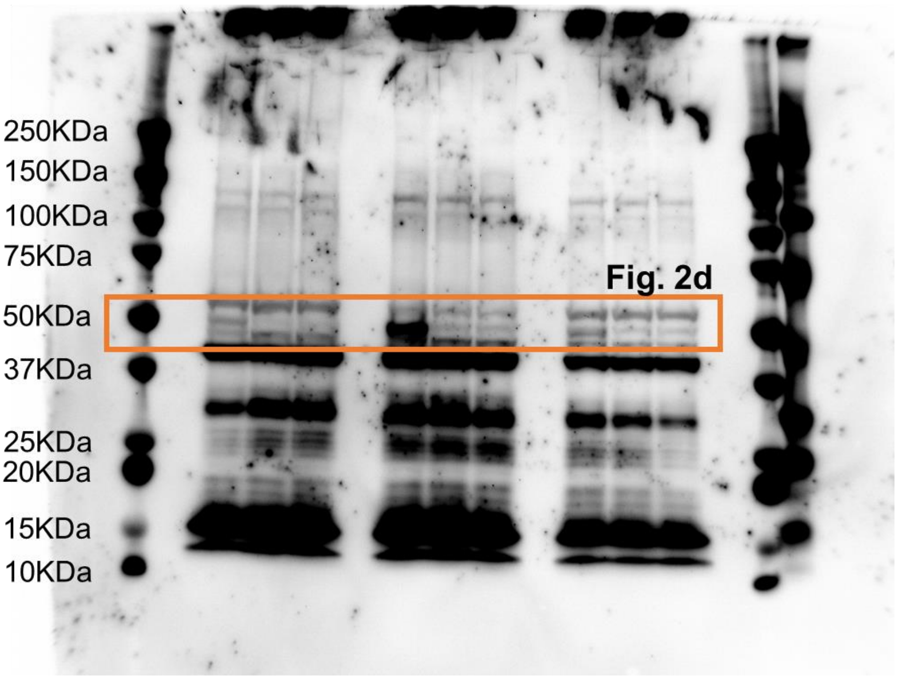

Original Image of β-actin Western Blotting gel

Using the same gel of p53 Western blotting, washed and then re-stained with anti- β-actin antibiody.

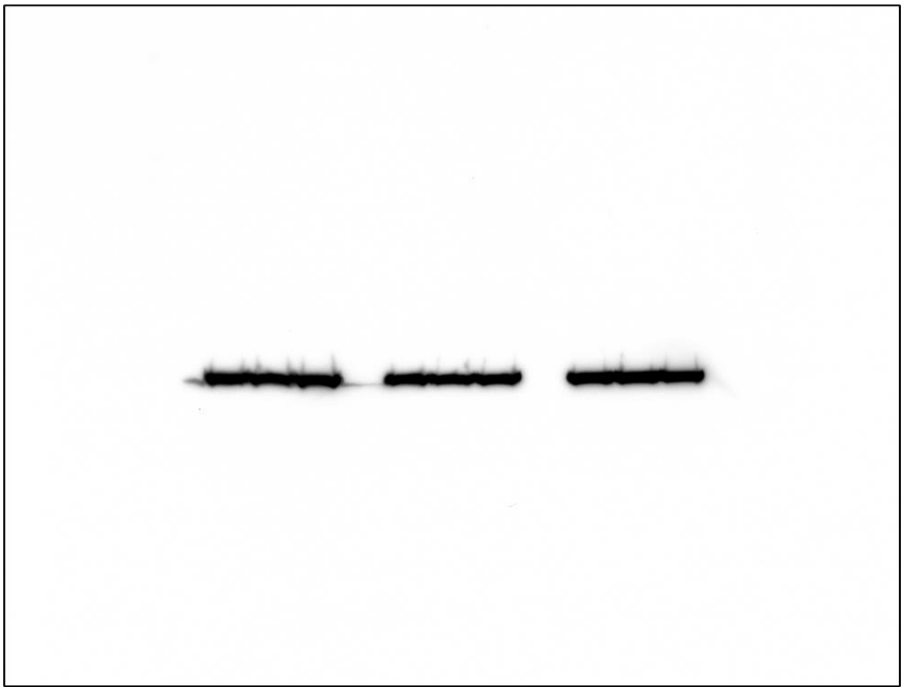

Supplement: Supplementary file 1 — Supplementary Information. [file 41598_2020_74660_MOESM1_ESM.pdf]
